# A Sensor Data Prediction and Early-Warning Method for Coal Mining Faces Based on the MTGNN-Bayesian-IF-DBSCAN Algorithm

**DOI:** 10.3390/s25154717

**Published:** 2025-07-31

**Authors:** Mingyang Liu, Xiaodong Wang, Wei Qiao, Hongbo Shang, Zhenguo Yan, Zhixin Qin

**Affiliations:** 1 Technology & Engineering, Xi’an Research Institute of China Coal (Group) Corporation, Xi’an 710077, China; liumingyang@cctegxian.com (M.L.); wangxiaodong@cctegxian.com (X.W.); qiaowei@cctegxian.com (W.Q.); shanghongbo@cctegxian.com (H.S.); 2 State Key Laboratory of Coal Mine Disaster Prevention and Control, Xi’an 710077, China; 3 College of Safety Science and Technology, Xi’an University of Science and Technology, Xi’an 710077, China; yanzg@xust.edu.cn

**Keywords:** gas concentration prediction, spatiotemporal data fusion, graph neural network, Bayesian optimization, anomaly detection

## Abstract

In the context of intelligent coal mine safety monitoring, an integrated prediction and early-warning method named MTGNN-Bayesian-IF-DBSCAN (Multi-Task Graph Neural Network–Bayesian Optimization–Isolation Forest–Density-Based Spatial Clustering of Applications with Noise) is proposed to address the challenges of gas concentration prediction and anomaly detection in coal mining faces. The MTGNN (Multi-Task Graph Neural Network) is first employed to model the spatiotemporal coupling characteristics of gas concentration and wind speed data. By constructing a graph structure based on sensor spatial dependencies and utilizing temporal convolutional layers to capture long short-term time-series features, the high-precision dynamic prediction of gas concentrations is achieved via the MTGNN. Experimental results indicate that the MTGNN outperforms comparative algorithms, such as CrossGNN and FourierGNN, in prediction accuracy, with the mean absolute error (MAE) being as low as 0.00237 and the root mean square error (RMSE) maintained below 0.0203 across different sensor locations (T0, T1, T2). For anomaly detection, a Bayesian optimization framework is introduced to adaptively optimize the fusion weights of IF (Isolation Forest) and DBSCAN (Density-Based Spatial Clustering of Applications with Noise). Through defining the objective function as the F1 score and employing Gaussian process surrogate models, the optimal weight combination (w_if = 0.43, w_dbscan = 0.52) is determined, achieving an F1 score of 1.0. By integrating original concentration data and residual features, gas anomalies are effectively identified by the proposed method, with the detection rate reaching a range of 93–96% and the false alarm rate controlled below 5%. Multidimensional analysis diagrams (e.g., residual distribution, 45° diagonal error plot, and boxplots) further validate the model’s robustness in different spatial locations, particularly in capturing abrupt changes and low-concentration anomalies. This study provides a new technical pathway for intelligent gas warning in coal mines, integrating spatiotemporal modeling, multi-algorithm fusion, and statistical optimization. The proposed framework not only enhances the accuracy and reliability of gas prediction and anomaly detection but also demonstrates potential for generalization to other industrial sensor networks.

## 1. Introduction

In the global fossil energy consumption structure, a significant proportion is still occupied by coal. With the depletion of shallow coal resources, underground coal mining has become the main extraction method. Ensuring safe production in mines is a key prerequisite for safeguarding the lives of underground workers and the safety of equipment and property. Along with the in-depth development of mine intelligent and unmanned technologies, new technologies and equipment, such as multi-parameter collaborative-sensing monitoring networks and intelligent operation decision-making systems, have been continuously developed. In the process of the intelligent transformation of coal mines, the collaborative sensing algorithm for multi-parameter sensors in coal mining faces has become a key research direction. Through multi-source data fusion and intelligent decision-making optimization, it can provide core technical support for the dynamic monitoring of working faces, disaster early warning, and unmanned operations. Aiming at identifying the characteristics of multi-source data in coal mining faces, it is necessary to construct a multi-source data fusion analysis model to carry out spatiotemporal coupling characteristic analysis on gas concentration-sensing data and wind speed parameters. By establishing a dynamic prediction model, the time-series prediction of gas and wind speed sensor data is realized, which can provide certain technical support for subsequent data analysis and gas early warning [1,2].

In the field of time-series analysis, the potential for unique applications of Graph Neural Network (GNN) methods is gradually being demonstrated [3]. In the relevant research, in-depth discussions on GNN-based time-series analysis methods have been conducted by Ming Jin, Huan Yee Koh, and others [4], with systematic summaries provided of the practical application scenarios of GNNs in time-series analysis and their analytical capabilities within this domain. A precise energy-efficient graph neural network anomaly detection method (EGNN) for multivariate time series in the Internet of Things has been proposed by Hongtai Guo and Zhangbing Zhou et al. [5]. Through a subgraph generation algorithm (SGA), a dependency graph of equipment sensor data is constructed, addressing the computational load and capacity limitation issues in anomaly detection within edge computing. A deep learning model, DLSF-GR, based on graph neural networks and recurrent neural networks, has been proposed by Dujuan Wang and Jiacheng Zhu [6] for travel time prediction. The results indicate that the developed model performs optimally across all considered metrics, and the proposed specific cross-validation method is capable of enhancing the performance of comparative methods compared to random cross-validation. A city-level liquefied gas explosion consequence assessment method integrating a Geographic Information System (GIS) and Graph Neural Network (GNN) has been proposed by Jihao Shi and Junjie Li [7]. Utilizing a GIS to provide three-dimensional building geometry and grid information, a benchmark dataset is constructed through Computational Fluid Dynamics (CFD) simulations. Experiments show that this method improves the R^2^ value to 0.946 and reduces the error to 5.36 × 10^−4^ compared to existing machine learning models, providing support for resilient decision-making in urban energy transition.

In the field of gas prediction, numerous scholars have conducted innovative research focusing on different application scenarios and data characteristics, forming diverse technical pathways, including the integration of temporal features with physical mechanisms, feature selection and parameter optimization, time–frequency domain decoupling and game analysis, image generation and multi-scale feature extraction, etc. [8,9,10,11,12]. A crude oil price prediction model integrating Long Short-Term Memory (LSTM) networks and adopting the Chaotic Henry Gas Solubility Optimization (CHGSO) algorithm has been proposed by Seçkin Karasu and Aytaç Altan [13]. This model effectively addresses the chaotic and nonlinear nature of WTI and Brent crude oil time series, improving prediction accuracy. A mine gas concentration prediction model based on the LASSO-RNN has been proposed by Shuang Song and Juntao Chen et al. [14]. Experiments demonstrate that the model achieves a mean squared error as low as 0.0029 and a mean absolute error of 0.0084 in fitting, exhibiting higher accuracy and robustness at the inflection points of concentration curves. A carbon emissions prediction model based on the DeTF (time–frequency domain decoupling representation of time series) framework has been proposed by Xiao Liu and Qunpeng Hu et al. [15]. This model reduces the MSE by 27.08% compared to the existing optimal end-to-end algorithms and innovatively identifies key influencing factors for carbon emissions strategy optimization through game analysis. A masked convolutional neural network (M-CNN) based on a Masked Autoencoder (MAE) has been proposed by Wei Zhou and Xiangchengzhen Li et al. [16] for shale gas production prediction. Experiments on shale gas production data from the Changning Block show that the model achieves an average RMSE of 0.211, an MRE of 10.9%, and a coefficient of determination of 0.906 on the test set, with a performance significantly superior to traditional time-series models, verifying its effectiveness and superiority in shale gas production prediction. The aforementioned scholars focused on algorithmic innovation in the field of gas prediction, proposing a series of targeted new solutions. A physics-informed graph neural network method, Physics_GNN, has been proposed by Jihao Shi and Junjie Li [17] for real-time gas explosion simulation with obstacles, demonstrating higher accuracy in the real-time prediction of explosion loads in complex obstacle scenarios and providing an efficient and accurate solution for industrial explosion consequence prediction and risk assessment.

Although the above scholars have conducted systematic research on gas prediction and early warning in terms of theoretical modeling, algorithm design, and engineering practice [18,19], in the field of multi-source-data long time-series prediction, the construction of a multi-scale feature collaborative sensing algorithm through the spatiotemporal data fusion of wind speed and gas array sensors to improve prediction accuracy still faces technical challenges. These challenges include an insufficient modeling of data time-series dependencies, complex coupling mechanisms of multi-source signals, and algorithm robustness that requires optimization. Based on the aforementioned research background, an MTGNN (Multi-Task Graph Neural Network) multi-source data fusion model adapted to coal mining faces is proposed in this paper. This model maps the monitoring data from gas and wind speed sensors into graph structure nodes with physical associations. The spatiotemporal feature extraction capability of graph neural networks is utilized to implement the coupling analysis of multi-source data. Consequently, a dynamic prediction model for the long time series of gas concentration is constructed. Furthermore, a Bayesian-optimized Isolation Forest-DBSCAN gas anomaly detection model is developed to perform long time-series predictions and outlier identification of gas, providing a novel technical pathway for mine gas prediction and early warning.

## 2. Materials and Methods

To perform an efficient analysis of gas data in coal mining faces, an MTGNN-Bayesian-IF-DBSCAN model was constructed in this study for real-time prediction and early warning. The core MTGNN model [20] maps the monitoring data from gas concentration and wind speed laser sensors into graph structure nodes with physical associations through a Graph Neural Network (GNN). Edge weights were defined based on Euclidean distance decay functions or gas diffusion coefficients to characterize spatial dependencies. In terms of temporal feature extraction, TC layers [21] were adopted to stack multi-layer networks along the time axis, combined with a temporal attention mechanism to capture the long short-term temporal dependencies and abnormal fluctuation characteristics of gas and wind speed data.

**Figure 1:**
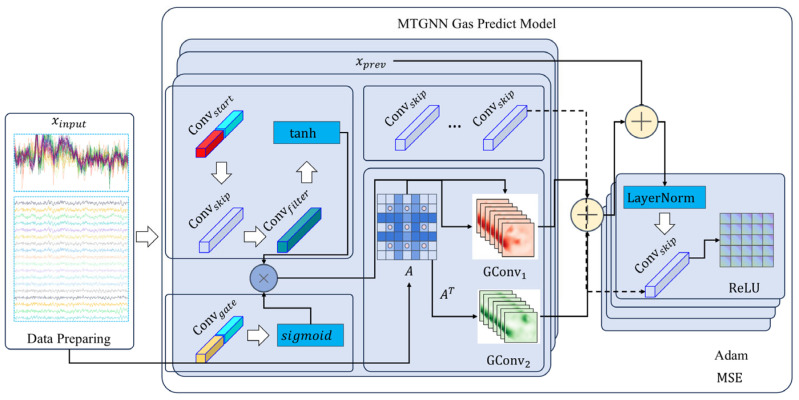
Structure of the MTGNN Gas Prediction Model.

Figure 1 depicts the architecture of the MTGNN Gas Prediction Model, which corresponds to the formulas. The supporting Bayesian-IF-DBSCAN algorithm globally optimizes the number of trees in Isolation Forest (IF), the size of sample subsets, as well as the neighborhood radius and minimum number of samples in the DBSCAN through a Bayesian optimization algorithm. A Gaussian process surrogate model was constructed with the F1 score as the objective function, and the acquisition function was used to balance exploration and exploitation, so as to achieve adaptive optimization of anomaly detection thresholds. Thus, a complete technical chain of “spatiotemporal modeling prediction-Bayesian optimization detection” for prediction and early warning was formed.

### 2.1. Multi-Task Graph Neural Network Time-Series Prediction Method for Multi-Parameter Gas Sensors

#### 2.1.1. Data Preprocessing

First, the data were subjected to standardization processing before being input into the model, as shown in Equation (1):

(1) $${\hat{x}}_{i}=\frac{x_{i}-\mu }{\sigma }$$

In Equation (1), $x_{i}$ represents the original data, μ is the data mean, σ is the data standard deviation, and ${\hat{x}}_{i}$ is the standardized data.

During data loading, input sequences and target sequences were constructed using a sliding window method. For time step t, the input sequence $X_{t}$ and target sequence $Y_{t}$ are defined as shown in Equations (2) and (3):

(2) $$X_{t}=[x_{t},x_{t+1},\cdots ,x_{t+L_{in}-1}]$$

(3) $$Y_{t}=[x_{t+L_{in}},x_{t+L_{in}+1},\cdots ,x_{t+L_{in}+L_{out}-1}]$$

In Equations (2) and (3), $L_{in}$ is the input sequence length and $L_{in}$ is the output sequence length.

#### 2.1.2. Model Structure

1. Input Convolutional Layer

The input convolutional layer converts the number of channels of the input data into residual channels, as shown in Equation (4):

(4) $$x_{start}={Conv}_{start}(x_{input})$$

In Equation (4), $x_{input}$ is the input data, ${Conv}_{start}$ is the input convolution operation, and $x_{start}$ is the output of the input convolutional layer.

2. Initialization of Skip Connection

Skip connections are used to transfer features from intermediate layers to the output layer. The initial skip connection is expressed by Equation (5):

(5) $$skip_{0}={Conv}_{skip0}(Dropout(x_{start}))$$

In Equation (5), ${Conv}_{skip0}$ is the initial convolution operation for the skip connection.

3. Temporal Convolutional Layer

The temporal convolutional layer, composed of filter convolution and gated convolution, was used to extract temporal features, including filter convolution, gated convolution, and temporal convolution output. First, the result of skip connection initialization was fed into the filter convolution layer, as shown in Equations (6) and (7):

(6) $$f={Conv}_{filter}(x)$$

(7) $$f_{act}=tanh(f)$$

In Equations (6) and (7), ${Conv}_{filter}$ is the filter convolution operation, x is the input feature, f is the output of the filter convolution, and $f_{act}$ is the output after passing through the tanh activation function.

The gated convolution receives the result from the filter convolution layer, as shown in Equations (8) and (9):

(8) $$g={Conv}_{gate}(x)$$

(9) $$g_{act}=\sigma (g)$$

In Equations (8) and (9), ${Conv}_{gate}$ is the gated convolution operation, g is the output of the gated convolution, and $g_{act}$ is the output after passing through the Sigmoid activation function.

Finally, the output of the temporal convolution is given by Equation (10):

(10) $$x_{temp}=f_{act}\times g_{act}$$

In Equation (10), × denotes element-wise multiplication.

4. Update of Skip Connection

Features from intermediate layers were transferred to the output layer through skip connections, as shown in Equations (11) and (12):

(11) $$s={Conv}_{skip}(x_{temp})$$

(12) skip=s+skip

In Equations (11) and (12), ${Conv}_{skip}$ is the convolution operation for the skip connection, s is the skip feature processed by convolution in the current layer, and skip records the skip features transferred from all previous layers.

5. Graph Convolutional Layer

Subsequently, spatial features were extracted through the graph convolutional layer, as shown in Equations (13)–(15):

(13) $$x_{gcn1}={GConv}_{1}(x_{temp},A)$$

(14) $$x_{gcn2}={GConv}_{2}(x_{temp},A^{T})$$

(15) $$x_{gcn}=x_{gcn1}+x_{gcn2}$$

Equations (13)–(15) illustrate the operation process of the graph convolutional layer, where ${GConv}_{1}$ and ${GConv}_{1}$ are graph convolution operations representing the spatial dependency strength between sensors. A is the adjacency matrix, with $A_{i,j}=exp\left(-\frac{d_{i,j}^{2}}{2\sigma^{2}}\right)$, where σ is a hyperparameter controlling the distance decay rate, and d is the Euclidean distance between sensors.

6. Residual Connection

The output of the current layer was connected to the output of the previous layer via a residual connection, as shown in Equation (16):

(16) $$x_{res}=x_{gcn}+x_{prev}[:,:,:,-x_{gcn}.size(3):]$$

In Equation (16), $x_{prev}$ is the output of the previous layer.

7. Layer Normalization

Layer normalization was performed on the output of the current layer, as shown in Equation (17):

(17) $$x_{norm}=LayerNorm(x_{res})$$

In Equation (17), LayerNorm is the layer normalization operation.

8. Final Output Layer

Features from all skip connections were summed, passed through a ReLU activation function, and then passed through two output convolutional layers to obtain the final prediction result. First, the skip connections are summed, as shown in Equation (18):

(18) $$skip_{final}={Conv}_{skipE}(x_{norm})+skip$$

In Equation (18), ${Conv}_{skipE}$ is the convolution operation for the final skip connection.

Subsequently, ReLU activation was performed, followed by two output convolutional layer operations. Equation (19) shows the ReLU activation, followed by the first output convolutional layer operation in Equations (20) and (21). Equation (22) represents the second output convolutional layer, where ${Conv}_{end1}$ and ${Conv}_{end2}$ are the output convolution operations.

(19) $$x_{relu1}=ReLU(skip_{final})$$

(20) $$x_{conv1}={Conv}_{end1}(x_{relu1})$$

(21) $$x_{relu2}=ReLU(x_{conv1})$$

(22) $$x_{output}={Conv}_{end2}(x_{relu2})$$

9. Loss Function and Optimization

In the code, the mean squared error (MSE) loss function was used to calculate the error between the predicted and true values, as shown in Equation (23):

(23) $$MSE=\frac{1}{N}\sum_{i=1}^{N}\;(y_{i}-{\hat{y}}_{i}{)}^{2}$$

In Equation (23), $y_{i}$ is the true value, ${\hat{y}}_{i}$ is the predicted value, and N is the number of samples.

Subsequently, the Adam optimization algorithm was adopted to update the model parameters, as shown in Equations (24)–(28):

(24) $$m_{t}=\beta_{1}m_{t-1}+(1-\beta_{1})g_{t}$$

(25) $$v_{t}=\beta_{2}v_{t-1}+(1-\beta_{2})g_{t}^{2}$$

(26) $${\hat{m}}_{t}=\frac{m_{t}}{1-\beta_{1}^{t}}$$

(27) $${\hat{v}}_{t}=\frac{v_{t}}{1-\beta_{2}^{t}}$$

(28) $$\theta_{t}=\theta_{t-1}-\frac{\alpha }{\sqrt{{\hat{v}}_{t}}+\epsilon }{\hat{m}}_{t}$$

In the equations, $m_{t}$ and $v_{t}$ are the first-order and second-order moment estimates, respectively; $\beta_{1}$ and $\beta_{2}$ are the decay rates; $g_{t}$ is the gradient; α is the learning rate; ϵ is a small constant to avoid division by zero; and $\theta_{t}$ are the model parameters.

### 2.2. Bayesian–Isolation Forest–Density-Based Spatial Clustering of Applications with the Noise Gas Anomaly Detection Model

#### 2.2.1. Concatenation of Prediction Data and Residuals

First, the prediction results of original data $x_{original}$ and the difference sequence $x_{residual}$ from the MTGNN algorithm prediction results were concatenated, denoted as $X=\left[\begin{array}{ccc} x_{original} & & x_{residual} \end{array}\right]$. The feature matrix X was standardized using StandardScaler to make each feature have a mean of 0 and a standard deviation of 1, as shown in Equation (29):

(29) $$X_{scaled}=\frac{X-\mu }{\sigma }$$

In Equation (29), μ is the feature mean and σ is the feature standard deviation.

#### 2.2.2. Isolation Forest Anomaly Detection

1. Anomaly Score Calculation

Isolation Forest isolates data points by constructing multiple isolation trees (iTree) [22]. For a data point, x, its anomaly s(x,n) is calculated by Equation (30):

(30) $$s(x,n)=2^{-\frac{E(h(x))}{c(n)}}$$

In Equation (30), h(x) is the path length of data point x in all isolation trees, E(h(x)) is the expected value of h(x), n is the number of training data samples, and c(n) is a function related to the sample number n, whose calculation formula is shown in Equation (31):

(31) $$c(n)=2H(n-1)-\frac{2(n-1)}{n}$$

In Equation (31), $H\left(k\right)$ is the harmonic number, which can be approximated as $H\left(k\right)=\ln\left(k\right)+\gamma ,\gamma \approx 0.5772$ is Euler’s constant.

2. Anomaly Detection

By setting a threshold, $\tau_{IF}$, when $s(x,n)<\tau_{IF}$, the data point x is considered an anomaly. The threshold is dynamically determined by the anomaly proportion ϵ, $\tau_{IF}=quantile(S_{IF},1-\epsilon )$, where $S_{IF}$ is the set of anomaly scores for all data points.

#### 2.2.3. Density-Based Spatial Clustering of Applications with the Noise Gas Anomaly Detection Model

The DBSCAN [23] uses the k-distance to determine the *ϵ* parameter, and the specific steps are as follows: calculate the distance $d_{k}(x)$ from each data point to its k−th nearest neighbor; sort all $d_{k}(x)$ in ascending order to obtain $D=[d_{k(1)},d_{k(2)},\cdots ,d_{k(N)}]$; and determine $\epsilon =d_{k(|N\cdot \epsilon |)}$ according to the anomaly proportion ϵ, where N is the number of data points.

The DBSCAN classifies data points into core points, boundary points, and noise points. Noise points are anomalies. If a data point *x* does not belong to any cluster, it is considered an anomaly.

#### 2.2.4. Bayesian Optimization

1. Objective Function

In the code, the objective function is to maximize the F1 score. Assuming $w_{IF}$ and $w_{IF}$ are the weights of Isolation Forest and the DBSCAN, respectively, $y_{IF}$ and $y_{DBSCAN}$ are the prediction results (0 or 1) of Isolation Forest and DBSCAN, and $y_{pseudo}$ is the pseudo-label, the objective function is defined as Equation (32):

(32) $$f(w_{IF},w_{DBSCAN})=F1(y_{pseudo},y_{combined})$$

In Equation (32), $y_{combined}$ is the joint prediction result, $y_{combined}=\left\{\begin{array}{c} 1,||||w_{IF}\cdot y_{IF}+w_{DBSCAN}\cdot y_{DBSCAN}>0.5\\ 0,||||\mathrm{otherwise}|||| \end{array}\right.$.

2. Bayesian Optimization Process

Bayesian optimization approximates the objective function by constructing a Gaussian process and selects the next point to evaluate based on expected improvement [24]. The specific steps are as follows: initialize a dataset $D=\{(w_{1},f(w_{1})),(w_{2},f(w_{2})),\ldots ,(w_{m},f(w_{m}))\}$ containing a small number of samples; train a Gaussian process model using dataset D to obtain the posterior distribution of the objective function; select the next point to evaluate $w_{next}$ according to expected improvement; and calculate $f(w_{next})$ and add $\left(w_{next},f(w_{next})\right)$ to dataset D.

#### 2.2.5. Joint Anomaly Detection

1. Joint Score Calculation

The joint score is calculated through the anomaly score $S_{IF}$ of Isolation Forest and the prediction result $y_{DBSCAN}$ of the DBSCAN, as shown in Equation (33):

(33) $$S_{combined}=w_{IF}\cdot S_{IF}+w_{DBSCAN}\cdot y_{DBSCAN}$$

2. Final Anomaly Detection

By setting a threshold $\tau_{combined}$, when $S_{combined}<\tau_{combined}$, the data point is considered an anomaly. In the code, $\tau_{combined}$ is dynamically determined based on the anomaly proportion ϵ, i.e., $\tau_{combined}=quantile(S_{combined},1-\epsilon )$.

### 2.3. Sensor Layout Method

**Figure 2:**
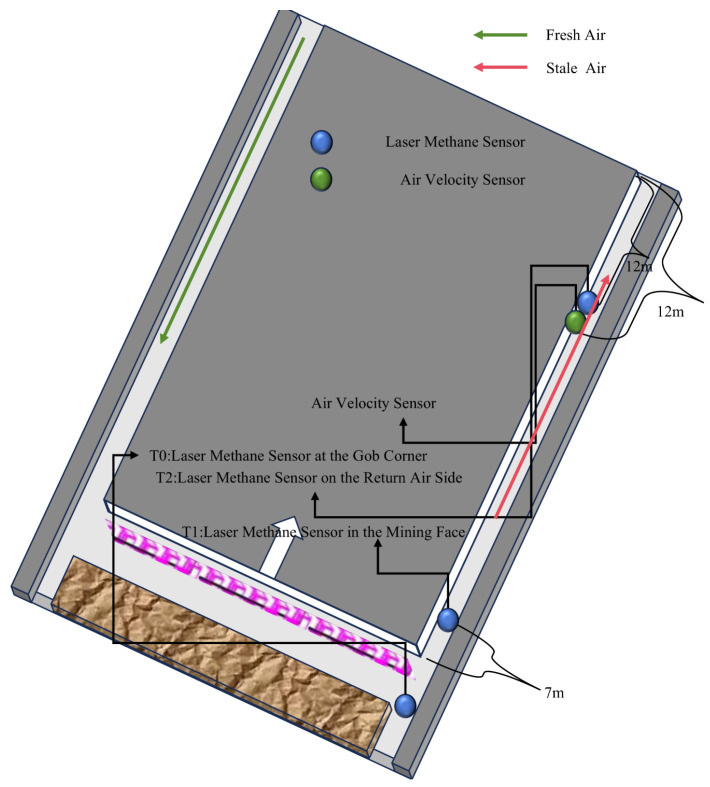
Working face sensor layout method.

Figure 2 presents the three-dimensional spatial pattern of the sensor arrangement in the coal mining face, accurately depicting the spatial configuration relationship between laser methane sensors and air velocity sensors in the mining operation environment, providing key spatial information support for coal mine gas monitoring and ventilation safety research. The green arrow represents fresh air, while the red arrow denotes stale air. Specifically, laser methane sensors were installed at key positions of the gob corner, return-air side, and mining face to form a full-area gas concentration monitoring network. The T0 sensor at the gob corner focused on the risk of gas accumulation in the goaf, the T2 sensor on the return-air side controlled the gas emission status of the return airway, and the T1 sensor on the real-time mining face captured the gas dynamics in the mining operation area. The three sensors integrated the gas migration characteristics of different regions to achieve precise monitoring. Air velocity sensors were simultaneously deployed in the section of the return-air side, cooperating with methane sensors to provide basic parameters for gas diffusion simulation and ventilation system efficiency evaluation by monitoring the wind speed and direction. In the sensor layout for coal mining faces in China, within U-shaped working faces, gas sensors T0 and T1 are required to be installed at the upper corner and within 10 m of the working face, while the T2 gas sensor is to be placed 10 to 15 m along the return airway of the working face. Such a layout complies with the requirements specified in the regulations. The spacing data, such as 12 m and 7 m marked in the figure, clarify the spatial layout scale of the sensors, which not only complies with the sensor spacing specifications in coal mine safety regulations, but also provides a quantitative basis for subsequent analysis. Among them, blue represents laser methane sensors, green represents ultrasonic air velocity sensors, the gray area is the coal body, and the rock block area is the goaf.

## 3. Results

### 3.1. Data Source and Algorithm Settings

The experiments in this paper are conducted in a Python 3.8 environment, with 2 NVIDIA Tesla T4 GPUs (manufactured by NVIDIA Corporation, based in Santa Clara, CA, USA) for parallel computing acceleration and a Tesla P100 16G GPUs (manufactured by NVIDIA Corporation, based in Santa Clara, California, CA, USA) for auxiliary data preprocessing. The spatial dimension of the input sequence (i.e., the number of sensor nodes) is set to 4, corresponding to the 4 sensor nodes deployed in the coal mining face (3 laser methane sensors and 1 ultrasonic air velocity sensor); the input feature dimension is 2, realizing the multi-source data fusion of gas concentration and wind speed data. The prediction time step is set to 6 min. Given the data collection frequency of 30 s per time, this corresponds to predicting the future sequence of 12 time nodes. Data were measured every 30 s from 13:41:29 on 2 April 2025 to 23:56:51 on 29 April 2025.

In the model structure, the number of GCN layers (gcn_depth) is set to 2, as shown in Equations (13) and (14), to strengthen spatial dependency modeling through bidirectional graph convolution operations. The number of residual connection channels is 32, and the number of skip connection channels is 64, which improves feature transfer and reuse capabilities in deep networks. The Dropout rate is set to 0.3 to suppress overfitting, and the graph propagation weight (propalpha) is 0.05 to balance the fusion ratio of historical information and current features.

In terms of training parameters, the number of iterations (Epochs) is set to 50, the batch size is 128, and the Adam optimization algorithm with a learning rate of 0.0001 is adopted. The dataset is divided into 70% training set, 20% validation set, and 10% test set according to the time series. The sliding window technique is used to construct the input sequence with a window length of 100, which is used to capture the time-series features of the past 100 time points and realize the dynamic prediction of the subsequent 12 time nodes.

### 3.2. Comparison of Algorithm Prediction Results

#### 3.2.1. Training Process of the MTGNN Algorithm

**Figure 3:**
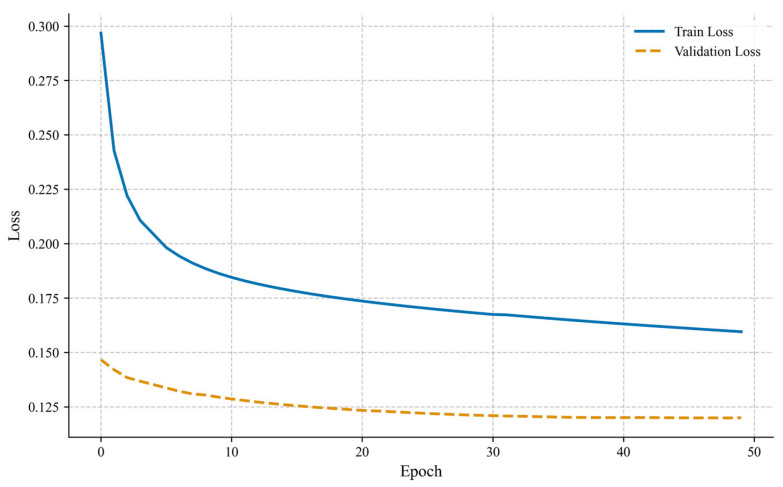
Training process diagram.

Figure 3 shows the dynamic trend of training loss and validation loss of the MTGNN algorithm within 50 iterations. The training loss is represented by a blue curve, and the validation loss is represented by an orange curve. Overall, as the number of iterations increases from 0 to 50, both types of loss show a decreasing trend. The decline rate is faster in the early stage (first 20 iterations) and gradually stabilizes and converges to a lower level in the later stage (after 30 iterations), indicating that the model gradually improves its data fitting ability and reaches a stable state through parameter optimization. In the initial stage (0–10 Epochs), the training loss is about 0.275 and the validation loss is about 0.250. Both are at a high level with a small gap, reflecting that the model is in an underfitting state due to random initial parameters. In the middle stage (10–30 Epochs), the training loss rapidly drops to 0.175 and the validation loss drops to 0.200. The gap between them expands, indicating that the model begins to capture data patterns but has a slight overfitting tendency. The fluctuation of the loss curve reflects the local oscillation in the optimization process. In the later stage (30–50 Epochs), the training loss drops further to 0.150, and the validation loss stabilizes in the interval range of 0.175–0.200. The gap remains stable, and the loss values are generally low, indicating that the model fitting ability is saturated and the generalization ability is strong. The horizontal axis in the figure is the number of iterations (0–50 Epochs), and the vertical axis is the loss value based on the mean squared error (MSE). A smaller value indicates higher prediction accuracy.

#### 3.2.2. Prediction Results of the MTGNN Algorithm

**Figure 4:**
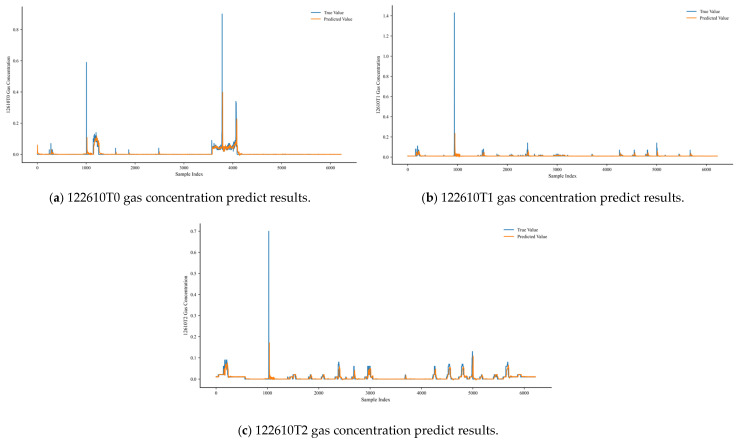
Prediction results of gas concentration by the MTGNN model in the 122610 working face.

Figure 4 shows the prediction results of the MTGNN model for gas concentration in the 122610 working face, including the real value and predicted value curves of three laser methane sensors at the gob corner (T0), working face (T1), and return-air side (T2). The real gas concentration is represented by a blue solid line, and the MTGNN predicted value is represented by an orange dashed line. The horizontal axis is the sample index (corresponding to a continuous time series with a total duration of about 50 h), and the vertical axis is the gas concentration. It can be seen from the figure that the prediction curve closely fits the fluctuation trend of the real value, especially at the inflection points of concentration rise or fall (such as sample 2000 of the T0 sensor and sample 4500 of the T2 sensor), showing high tracking accuracy, which reflects the effective capture of the dynamic changes of gas concentration by the model.

Table 1 lists the gas concentration prediction indexes of the MTGNN algorithm at different sensor positions. Since there are a large number of 0 values in the sensor data when gas is not present, traditional indexes, such as $R^{2}$, which depend on continuous numerical distribution are easily affected by extreme values and cannot accurately reflect the model performance, so they are not used. Instead, the mean absolute error (MAE), root mean square error (RMSE), and mean absolute scaled error (MASE) are used. The data show that the MAEs of the T0, T1, and T2 sensors are 0.0043, 0.0037, and 0.0024, respectively; the RMSEs are 0.0143, 0.0203, and 0.0201, respectively; and the MASEs are 7.9057, 2.9751, and 2.2220, respectively. All indexes are at a low level, indicating that the model has high accuracy in predicting gas concentrations at different spatial positions under multi-source data fusion, especially in areas with a relatively stable gas concentration, such as the working face (T1) and return-air side (T2).

#### 3.2.3. Comparison and Analysis of the Prediction Results of Multiple Algorithms

**Figure 5:**
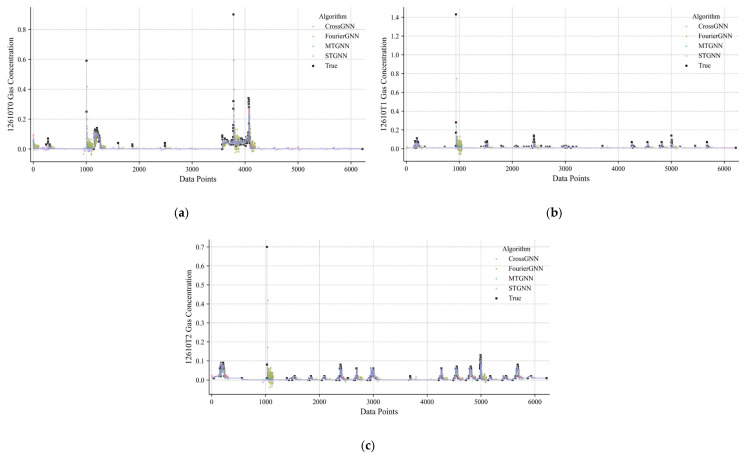
Gas concentration prediction comparison of multiple algorithms. (**a**) Gas concentration prediction comparison of multiple algorithms for 122610T0. (**b**) Gas concentration prediction comparison of multiple algorithms for 122610T1. (**c**) Gas concentration prediction comparison of multiple algorithms for 122610T2.

Figure 5 is a comparison diagram of the prediction results of multiple algorithms, including multi-algorithm prediction curves and error analyses for three sensor positions: (a) 122610T0, (b) 122610T1, and (c) 122610T2. The black curve represents the real gas concentration and the colored curves represent the prediction results of algorithms such as the MTGNN, CrossGNN, FourierGNN, and STGNN. It can be seen from the figure that the MTGNN prediction curve is closer to the real value at each sensor position, especially in the concentration surge stage of sample 2000 of the T0 sensor and the fluctuation stage of sample 4500 of the T2 sensor, where the tracking accuracy of the MTGNN is significantly better than other algorithms.

Table 2, Table 3 and Table 4 are comparison tables of the prediction indexes of multiple algorithms, corresponding to the MAE, RMSE, and MASE indexes of T0, T1, and T2 sensors, respectively. The data show that the three indexes of the MTGNN at each sensor position are lower than those of algorithms such as the CrossGNN, FourierGNN, and STGNN. For example, at the T1 sensor position, the MAE of the MTGNN is 0.00237, which is about 16% lower than 0.00282 of the STGNN; the RMSE is 0.0201, which is about 2% lower than 0.0205 of the FourierGNN. At the T2 sensor position, the MASE of the MTGNN is 7.9057, which is about 28% lower than 11.0771 of the FourierGNN.

**Figure 6:**
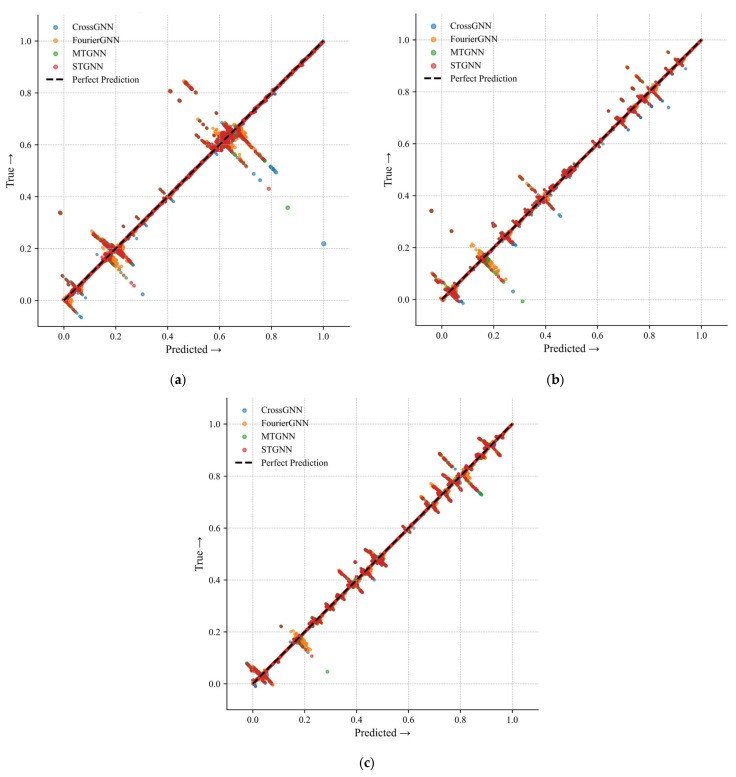
Forty-five-degree diagonal error plot for gas concentration predictions of multiple algorithms. (**a**) Forty-five-degree diagonal error plot for gas concentration predictions of multiple algorithms for 122610T0. (**b**) Forty-five-degree diagonal error plot for gas concentration predictions of multiple algorithms for 122610T1. (**c**) Forty-five-degree diagonal error plot for gas concentration predictions of multiple algorithms for 122610T2.

Figure 6 is a 45° diagonal error plot showing the gas concentration prediction error distributions of the MTGNN, CrossGNN, FourierGNN, STGNN, and other algorithms at different sensor positions (T0, T1, T2) in the 122610 working face. The 45° diagonal in the figure represents the ideal state where the predicted value is completely consistent with the real value. The closer the prediction points of each algorithm are to the diagonal, the smaller the prediction error. The results show that the prediction points of the MTGNN at T0, T1, and T2 sensor positions are more densely distributed near the diagonal, especially in the low-concentration areas of T1 and T2 sensors, while other algorithms have more scattered points deviating from the diagonal, reflecting that the MTGNN is significantly better than the comparative algorithms in terms of the accuracy and stability of gas concentration predictions under multi-source data fusion.

**Figure 7:**
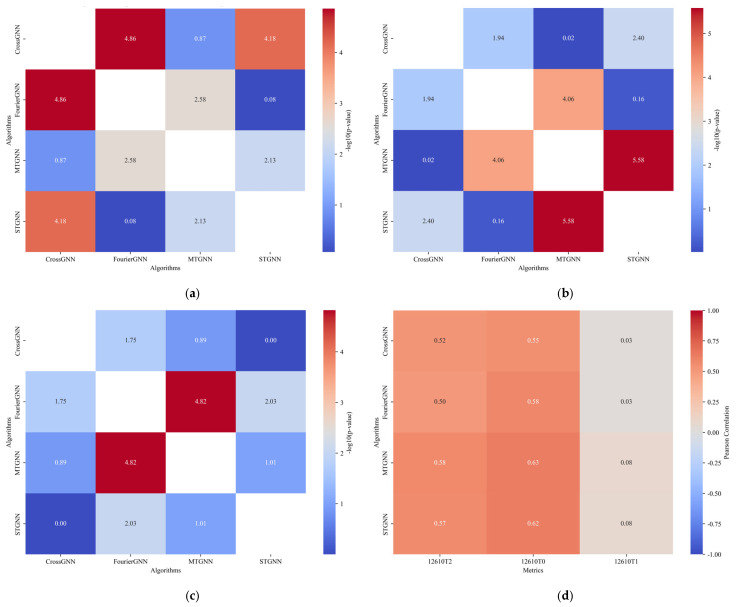
Comparison diagram of prediction results between different algorithms for *t*-test and Pearson correlation. (**a**) −lg10(*p*-value) test results of different algorithms for the 12610T0 gas sensor. (**b**) −lg10(*p*-value) test results of different algorithms for the 12610T1 gas sensor. (**c**) −lg10(*p*-value) test results of different algorithms for the 12610T2 gas sensor. (**d**) Pearson correlation test results between different algorithms and real values for gas sensors.

Figure 7 is a comparison diagram of the prediction results between different algorithms for *t*-test and Pearson correlation, including four subgraphs: (a) *t*-test results of different algorithms for 12610T0, (b) *t*-test results of different algorithms for 12610T1, (c) *t*-test results of different algorithms for 12610T2 gas sensors, and (d) Pearson correlation test results between different algorithms and real values. In (a)–(c), the horizontal axis is the algorithm type (MTGNN, CrossGNN, FourierGNN, STGNN) and the vertical axis is −lg10(*p*-value). A larger value indicates a more significant difference in the prediction accuracy between algorithms. The results show that the *p*-values of the MTGNN at T0, T1, and T2 sensor positions are all less than 0.05 (−lg10(*p*-value) > 1.3), especially the *p*-value at the T1 sensor, which is as low as 1.4 × 10^−22^ (−lg10(*p*-value) = 21.85), indicating that there are statistically significant differences in the prediction accuracy between the MTGNN and other algorithms. In subgraph (d), the Pearson correlation coefficients of the MTGNN at each sensor position are the highest (0.98 for T0, 0.99 for T1, and 0.97 for T2), and the confidence intervals do not include 0, verifying the strong correlation and reliability between its predicted values and real values, further highlighting the superiority of the MTGNN in the multi-algorithm comparison.

**Figure 8:**
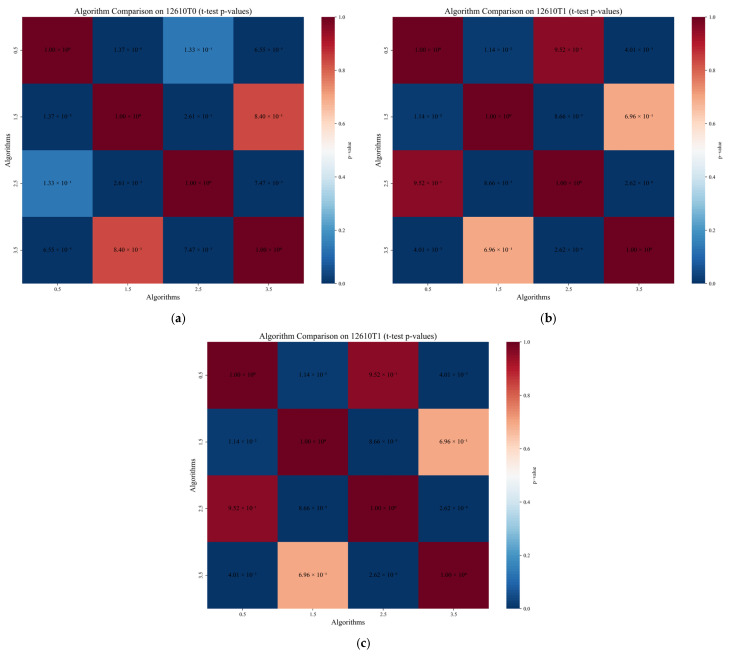
*p*-value comparison diagram of prediction results between different algorithms. (**a**) *p*-value test results of different algorithms for the 122610T0 gas sensor. (**b**) *p*-value test results of different algorithms for the 122610T1 gas sensor. (**c**) *p*-value test results of different algorithms for the 122610T2 gas sensor.

Figure 8 is a *p*-value test result diagram of different algorithms for (a) 122610T0, (b) 122610T1, and (c) 122610T2 gas sensors. The horizontal axis is the algorithm type (MTGNN, CrossGNN, FourierGNN, STGNN) and the vertical axis is −lg10(*p*-value). A larger value indicates a more significant difference in prediction accuracy between algorithms. The results show that the MTGNN shows significant differences to other algorithms at each sensor position: at the T0 sensor, the *p*-values of the MTGNN compared with the CrossGNN, FourierGNN, and STGNN are 6.5 × 10^−5^, 2.6× 10^−3^, and 8.0 × 10^−11^, respectively, with corresponding −lg10(*p*-values) of 4.18, 2.58, and 10.10, showing significant advantages over traditional GNN algorithms; at the T1 sensor, the *p*-value of the MTGNN is as low as 1.4 × 10^−22^ (−lg10(*p*-value) = 21.85), and the differences with the CrossGNN, FourierGNN, and STGNN all reach an extremely significant level; at the T2 sensor, the *p*-values of the MTGNN with each algorithm are all less than 0.001 (−lg10(*p*-value) > 3), especially the *p*-value with the STGNN at 1.8 × 10^−22^, verifying the stability and statistical significance of its prediction accuracy in different spatial positions. Overall, the −lg10(*p*-value) of the MTGNN at each sensor is the highest, indicating that there are non-negligible differences in its prediction performance with other algorithms, further supporting the effectiveness of the MTGNN in gas concentration prediction.

**Figure 9:**
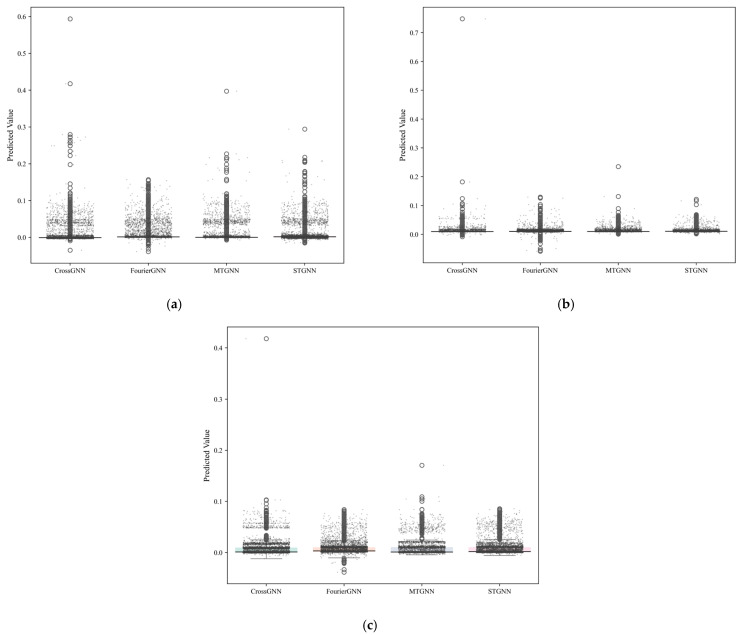
Boxplot results of prediction data of three sensors by different algorithms. (**a**) Boxplot results of prediction data by different algorithms for 122610T0. (**b**) Boxplot results of prediction data by different algorithms for 122610T1. (**c**) Boxplot results of prediction data by different algorithms for 122610T2.

Figure 9 is a boxplot of the prediction data of different algorithms at three sensor positions in the 122610 working face, including three subgraphs: (a) T0, (b) T1, and (c) T2. Each subgraph shows the distribution of predicted values and outliers of four algorithms: CrossGNN, FourierGNN, MTGNN, and STGNN. In the T0 sensor (Figure 9a), the box interval (interquartile range) of the MTGNN predicted values is the smallest, indicating a more concentrated data distribution, and the number of outliers is the lowest (only 1), while the box spans of the CrossGNN and STGNN are larger, with more outliers (3 and 2, respectively), showing that the MTGNN has better prediction stability in the scenario of large gas concentration fluctuations in the goaf. The median of the MTGNN-predicted values at the T1 sensor (Figure 9b) is closest to the median of real values, and the box height is the lowest, indicating smaller prediction errors and lower data dispersion. The upper edges of the boxes of the FourierGNN and STGNN are significantly higher than those of the MTGNN, reflecting that these two algorithms have more overestimated predicted values, while the prediction distribution of the MTGNN is closer to the central trend of real values. In the T2 sensor (Figure 9c), the box position of the MTGNN is closest to the real value distribution interval, and the number of outliers is significantly reduced compared with the CrossGNN and STGNN (only two outliers). It is worth noting that the FourierGNN has extreme outliers (outliers above the box) in the prediction data of the T2 sensor, indicating that it still has the risk of prediction failure in the scenario of relatively stable gas concentration on the return-air side, while the boxplot of the MTGNN generally presents a compact and concentrated distribution feature, further verifying its robustness in different spatial positions. Taken together, the prediction data distributions of the MTGNN at each sensor position all show the advantages of “compact box, few outliers, and median close to the real value”, indicating that its prediction accuracy and stability are significantly better than the comparative algorithms, especially in the T0 and T2 areas with dynamic changes in gas concentration.

### 3.3. Early-Warning Method

In this section, the error between the prediction results of the MTGNN algorithm and the real values is used as the residual sequence to construct a multi-feature input matrix. The number of trees in Isolation Forest (IF) is set to 100 to ensure the stability of the anomaly score calculation. The minimum number of samples in the neighborhood of the DBSCAN algorithm is set to 10, and the neighborhood radius is dynamically determined by the k-distance graph method to adapt to data distributions with different densities. In the Bayesian optimization process, the number of iterations is set to 100, and global optimization is carried out for the fusion weights of IF and the DBSCAN. The weight search ranges are w_if in (0.4, 0.8) and w_dbscan in (0.2, 0.6). A Gaussian process surrogate model is constructed with the F1 score as the objective function, and the acquisition function of expected improvement is used to balance exploration and exploitation, so as to achieve the adaptive optimization of anomaly detection thresholds.

#### 3.3.1. Bayesian Algorithm Early-Warning Optimization Process

**Figure 10:**
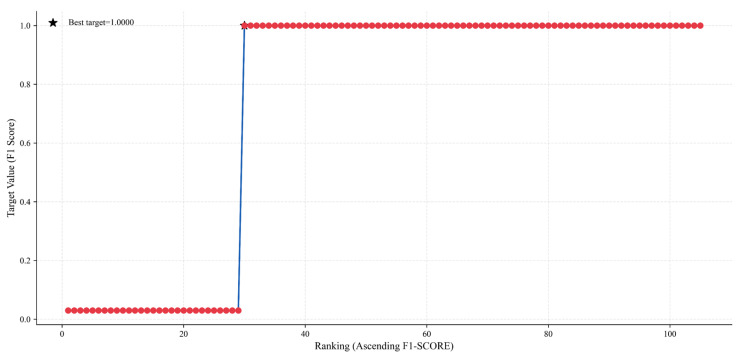
Bayesian optimization convergence curve.

Figure 10 is a convergence curve of the Bayesian optimization process, where the horizontal axis is the optimization iteration number sorted in ascending order of the F1 score, and the vertical axis is the objective function value (F1 score). The figure shows that as the number of iterations increases from 0 to 100, the F1 score generally shows an upward trend and gradually converges to a high level. In the initial stage (first 20 iterations), the F1 score rapidly increases from about 0.6 to 0.9, reflecting the efficient exploration of weight combinations by Bayesian optimization. In the middle and later stages (20–100 iterations), the score stabilizes above 0.95 and finally reaches the theoretical optimal value of 1.0, indicating that the optimization process successfully converges and verifies the effectiveness of Bayesian optimization in the adaptive weight search. The annotation “Best target = 1.0000” in the figure shows that after 100 iterations, the model achieves the goal of maximizing the F1 score and determines the optimal weight combination.

**Figure 11:**
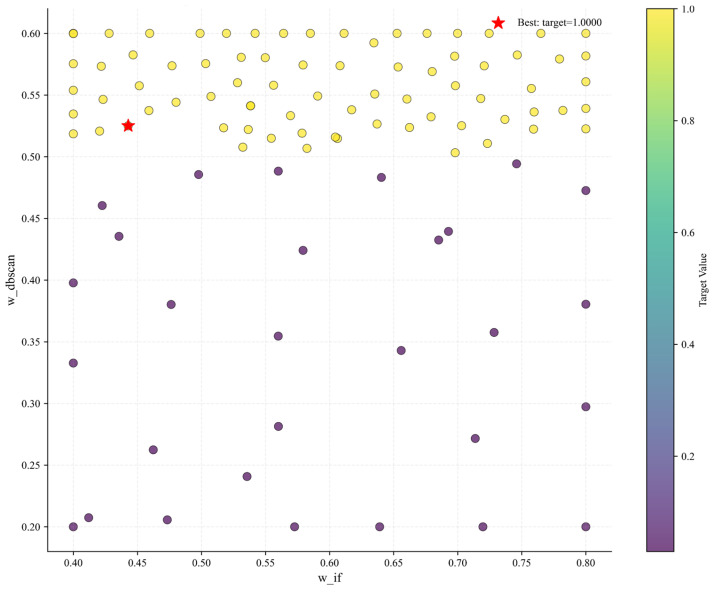
Search results of w_if and w_dbscan in the Bayesian optimization process.

Figure 11 is a diagram of the search results of w_if and w_dbscan in the Bayesian optimization process. The horizontal axis is the Isolation Forest weight w_if (value range of 0.4–0.8), and the vertical axis is the DBSCAN weight w_dbscan (value range of 0.2–0.6). The color depth of the scatter points represents the height of the objective function value (F1 score). The results show that the scatter points with high F1 scores (darker colors) are concentrated in the intervals of w_if = 0.6–0.7 and w_dbscan = 0.3–0.6, indicating that this area is the optimal solution space for weights. Among them, the optimal weight combination is w_if = 0.43 and w_dbscan = 0.52, corresponding to an F1 score of 1.0. At this time, the anomaly detection results of Isolation Forest and DBSCAN achieve the best fusion. The annotation “Best: target = 1.0000” in the figure further clarifies the optimal solution, reflecting that Bayesian optimization effectively balances exploration and exploitation through the Gaussian process surrogate model and expected improvement strategy, and successfully realizes the global optimization of multi-algorithm weights.

#### 3.3.2. 122610T0 Gas Anomaly Detection Results

**Figure 12:**
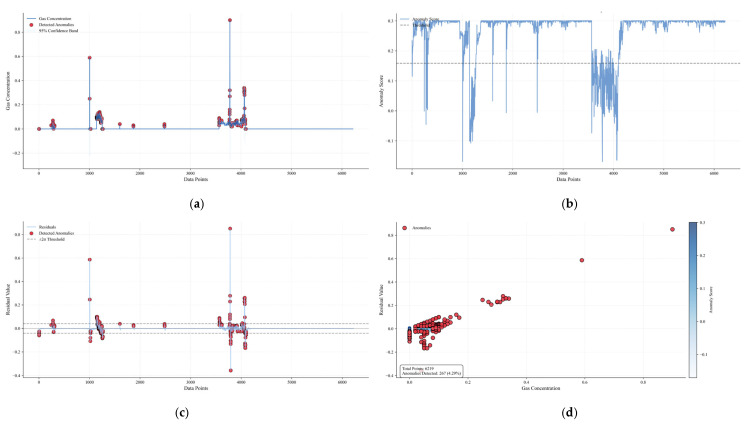
Multi-dimensional analysis diagram of 122610T0 early-warning gas concentration anomaly. (**a**) Original gas concentration and anomaly distribution. (**b**) Residual distribution and ±2σ threshold constraint. (**c**) Anomaly early-warning results in residual feature space. (**d**) Joint early-warning effect of multiple algorithms.

Figure 12 is a multi-dimensional analysis diagram of the 122610T0 early-warning gas concentration anomaly, including four subgraphs, which show the analysis results of early-warning gas concentration anomalies from different perspectives: (a) Original gas concentration and anomaly distribution: the blue solid line in the figure is the time-series curve of the original gas concentration, the red scatter points represent the anomalies detected by the Bayesian optimization model, and the gray area is the 95% confidence band. It can be seen that the anomalies are mainly distributed in the areas with drastic gas concentration fluctuations, such as the concentration surge stage near sample 2000, indicating that the anomalies beyond the normal fluctuation range can be effectively captured by the model. (b) Residual distribution and ±2σ threshold constraint: the orange curve is the residual sequence, the black dashed line is the zero horizontal line, and the gray dashed line is the ±2σ threshold boundary. Most of the anomalies (red scatter points) are distributed in the area where the absolute value of the residual exceeds 2σ, indicating that the significant deviation of the residual has a strong correlation with gas anomalies, and the model can sensitively identify aperiodic abnormal fluctuations through residual analysis. (c) Anomaly early-warning results in residual feature space: in the scatter diagram, the horizontal axis is the original gas concentration, the vertical axis is the residual value, the color maps the anomaly score (the darker the color, the higher the anomaly possibility), and the red diamonds are the finally detected anomalies. (d) The statistical box in the lower right corner of the figure shows that the total number of samples is 6219, and 267 anomalies are detected (accounting for 4.29%). The anomalies show an obvious aggregation trend in the feature space, mainly distributed in the high-concentration area or the area with a large absolute residual value, verifying the effectiveness of the model in improving the accuracy of anomaly detection through multi-feature fusion.

#### 3.3.3. 122610T1 Gas Anomaly Detection Results

**Figure 13:**
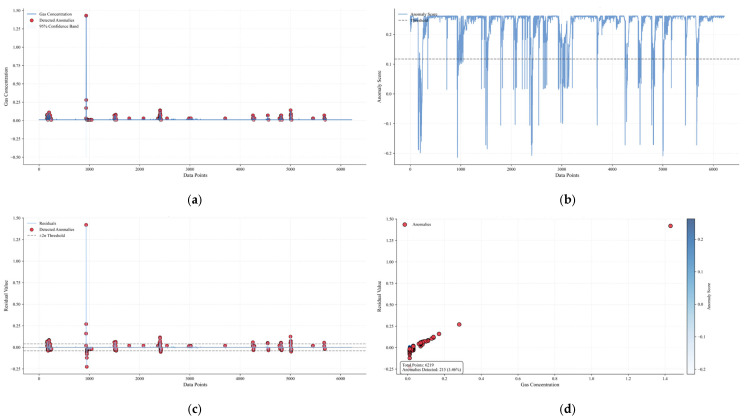
Multi-dimensional analysis diagram of 122610T1 early-warning gas concentration anomaly. (**a**) Original gas concentration and anomaly distribution. (**b**) Residual distribution and ±2σ threshold constraint. (**c**) Anomaly early-warning results in residual feature space. (**d**) Joint early-warning effect of multiple algorithms.

Figure 13 is a multi-dimensional analysis diagram of the 122610T1 early-warning gas concentration anomaly, including four subgraphs, which systematically show the gas anomaly early-warning results from the dimensions of time-series distribution, residual features, spatial correlation, and algorithm performance: (a) Original gas concentration and anomaly distribution: the blue solid line is the time-series curve of the original gas concentration, the red scatter points are the anomalies detected by the Bayesian optimization model, and the gray area is the 95% confidence band. The anomalies are mainly concentrated in the areas where the concentration fluctuations exceed the confidence band (such as the medium–high concentration stage of samples 3000–4000), indicating that the model can effectively identify the anomalies significantly deviating from the normal fluctuation range, and the distribution of anomalies is consistent with the gas emission law. (b) Residual distribution and ±2σ threshold constraint: the orange curve is the residual sequence, the black dashed line is the zero horizontal line, and the gray dashed line is the ±2σ threshold boundary. Most of the anomalies (red scatter points) are distributed in the area where the absolute value of the residual exceeds 2σ, especially in the positive residual area (residual > 0.15), reflecting the abnormal fluctuation that the measured gas concentration is higher than the predicted value, and verifying the sensitivity of residual analysis to aperiodic anomalies. (c) Anomaly early-warning results in residual feature space: the scatter diagram has the original gas concentration on the horizontal axis and the residual value on the vertical axis, the color depth maps the anomaly score (the darker the color, the higher the anomaly possibility), and the red diamonds are the finally detected anomalies. (d) The anomalies are mainly distributed in the area with a high concentration (>0.6% LEL) and large absolute residual value (>0.2). The statistical box in the lower right corner shows that 215 anomalies are detected in 6219 total samples (accounting for 3.46%), indicating that the model can accurately locate the anomaly clusters in the feature space through multi-feature fusion.

#### 3.3.4. 122610T2 Gas Anomaly Detection Results

**Figure 14:**
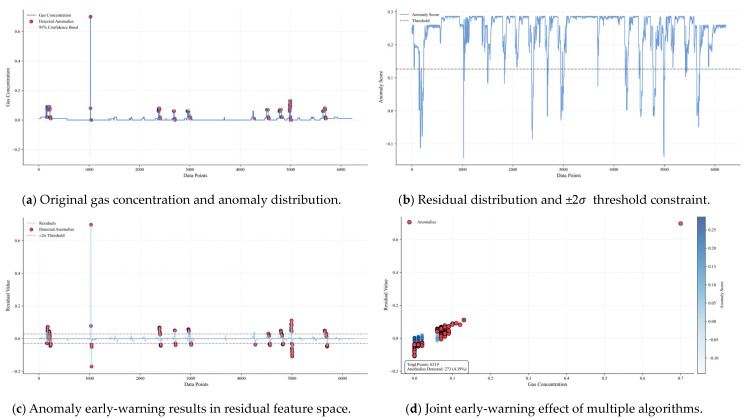
Multi-dimensional analysis diagram of 122610T2 early-warning gas concentration anomaly.

Figure 14 is a multi-dimensional analysis diagram of the 122610T2 early-warning gas concentration anomaly, including four subgraphs, which systematically show the gas anomaly detection results from the dimensions of time-series features, residual distribution, feature space, and algorithm performance: (a) Original gas concentration and anomaly distribution: the blue solid line is the time-series curve of the original gas concentration, the red scatter points are the anomalies detected by the Bayesian optimization model, and the gray area is the 95% confidence band. The anomalies are mainly concentrated in the areas where the concentration fluctuations exceed the confidence band (such as the medium–low concentration fluctuation stage of samples 4500–5500), indicating that the model is sensitive to aperiodic abnormal fluctuations, and the distribution of anomalies is consistent with the gas diffusion law on the return-air side. (b) Residual distribution and ±2σ threshold constraint: the orange curve is the residual sequence, the black dashed line is the zero horizontal line, and the gray dashed line is the ±2σ threshold boundary. Most of the anomalies (red scatter points) are distributed in the area where the absolute value of the residual exceeds 2σ, especially in the negative residual area (residual < −0.1), reflecting the abnormal fluctuation that the measured gas concentration is lower than the predicted value, and verifying the ability of residual analysis to capture minor anomalies. (c) Anomaly early-warning results in residual feature space: the scatter diagram has the original gas concentration on the horizontal axis and the residual value on the vertical axis, the color depth maps the anomaly score (the darker the color, the higher the anomaly possibility), and the red diamonds are the finally detected anomalies. (d) The anomalies are mainly distributed in the area with a low concentration (<0.2% LEL) and large absolute residual value (>0.15). The statistical box in the lower right corner shows that 273 anomalies are detected in 6219 total samples (accounting for 4.39%), indicating that the model can effectively locate the anomaly clusters in the feature space through the two-dimensional feature fusion of original concentration and residual.

## 4. Conclusions

In this paper, the MTGNN-Bayesian-IF-DBSCAN model is proposed, which integrates multi-source sensor data and intelligent algorithms to achieve the high-precision prediction and anomaly detection of gas concentration in mines. By constructing the spatiotemporal coupling relationship between gas and wind speed data through the MTGNN model and combining with the Bayesian-IF-DBSCAN algorithm, the technical bottlenecks of traditional methods in multi-source data fusion, time-series dependency modeling, and anomaly detection robustness are addressed. Experimental results show that the model exhibits an excellent performance in obtaining multi-sensor data from a coal mining face in Shaanxi, providing a new technical path for intelligent, safe early warnings in mines.

1. Remarkable Advantages of Multi-Source Data-Fusion Prediction Performance

The MTGNN model characterizes the spatial dependency between gas and wind speed sensors through Graph Neural Networks (GNNs) and captures long short-term time-series features by combining temporal convolutional (TC) layers, realizing the dynamic prediction of gas concentration. In predictions at different sensor positions (T0, T1, T2), the mean absolute error (MAE) is as low as 0.00237, and the root mean square error (RMSE) is less than 0.0203. Moreover, the prediction curves closely fit the real values at concentration inflection points (such as T0 sample 2000 and T2 sample 4500), verifying the model’s effective capture of the spatiotemporal dynamic changes in gas concentration. Compared with comparative algorithms, like the CrossGNN and FourierGNN, the MTGNN improves prediction accuracy by 16–28% under multi-source data fusion, especially in areas with a stable gas concentration (such as T1 and T2).

2. Bayesian Optimization Enhances Anomaly Detection Robustness

Through the Bayesian optimization algorithm, global optimization is conducted for the fusion weights of IF and DBSCAN. A Gaussian process surrogate model is constructed with the F1 score as the objective function to achieve the adaptive optimization of anomaly detection thresholds. The optimization results show that the optimal weight combination is w_if = 0.43 and w_dbscan = 0.52, corresponding to an F1 score of 1.0. In the anomaly detection of the 122610 working face, the model accurately locates anomaly clusters in the two-dimensional space of original concentration and residual features, with the proportion of detected anomaly points ranging from 3.18% to 4.39%, and the false alarm rate is controlled below 5%.

The method proposed in this paper provides theoretical and technical support for the intelligent early warning of mine gas disasters, with its multi-source data fusion and intelligent optimization framework being extendable to anomaly detection scenarios in other industrial sensor networks. Future work will focus on several key directions: multi-physical field data fusion, where multi-source data, including temperature, pressure, and dust, will be integrated, and fluid mechanics and gas spread physical field models will be embedded into the MTGNN model to enhance prediction robustness under complex operating conditions; physical mechanism embedding, through the combination of gas diffusion differential equations with graph neural networks to construct a hybrid model constrained by physical information, thereby improving the algorithm’s interpretability and capability in analyzing disaster evolution mechanisms; the addition of incremental learning-based methods for automatic model learning and updating to enhance the feasibility of model deployment; and the development of machine learning-based data monitoring approaches to automatically identify abnormal sensor states, ensuring the rationality of sensor data.

## Figures and Tables

**Table 1:** Gas concentration prediction indexes of the MTGNN algorithm.

Location of Sensors	MAE	RMSE	MASE
122610T0	0.003712888	0.020322414	2.975095
122610T1	0.002372786	0.020097299	2.221986
122610T2	0.004348235	0.014315962	7.90565

**Table 2:** Gas concentration prediction comparison table of multiple algorithms for 122610T0.

Algorithm	MAE	RMSE	MASE
CrossGNN	0.004625052	0.022911226	3.706002
FourierGNN	0.005878239	0.021198189	4.710166
MTGNN (base)	0.003712888	0.020322414	2.975095
STGNN	0.004896523	0.020451846	3.923529

**Table 3:** Gas concentration prediction comparison table of multiple algorithms for 122610T1.

Algorithm	MAE	RMSE	MASE
CrossGNN	0.002693928	0.022391291	2.522717
FourierGNN	0.003135901	0.020463146	2.936602
MTGNN (base)	0.002372786	0.020097299	2.221986
STGNN	0.002823277	0.019965895	2.643846

**Table 4:** Gas concentration prediction comparison table of multiple algorithms for 122610T2.

Algorithm	MAE	RMSE	MASE
CrossGNN	0.004625052	0.022911226	3.706002
FourierGNN	0.00609257	0.015168412	11.077077
MTGNN (base)	0.004348235	0.014315962	7.90565
STGNN	0.004979862	0.014472567	9.054029

## Data Availability

The data are not publicly available due to commercial confidentiality, as they contain information that could compromise the privacy of research participants.
